# Uremic Toxins and Their Relation with Oxidative Stress Induced in Patients with CKD

**DOI:** 10.3390/ijms22126196

**Published:** 2021-06-08

**Authors:** Anna Pieniazek, Joanna Bernasinska-Slomczewska, Lukasz Gwozdzinski

**Affiliations:** 1 Department of Molecular Biophysics, Faculty of Biology and Environmental Protection, University of Lodz, ul. Pomorska 141/143, 90-236 Lodz, Poland; joanna.bernsinska@biol.uni.lodz.pl; 2 Department of Pharmacology and Toxicology, Medical University of Lodz, 90-753 Lodz, Poland; lukasz.gwozdzinski@umed.lodz.pl

**Keywords:** chronic kdney disease, uremic toxins, oxidative stress, carbamylation, cardiovascular disease

## Abstract

The presence of toxins is believed to be a major factor in the development of uremia in patients with chronic kidney disease (CKD) and end-stage renal disease (ESRD). Uremic toxins have been divided into 3 groups: small substances dissolved in water, medium molecules: peptides and low molecular weight proteins, and protein-bound toxins. One of the earliest known toxins is urea, the concentration of which was considered negligible in CKD patients. However, subsequent studies have shown that it can lead to increased production of reactive oxygen species (ROS), and induce insulin resistance in vitro and in vivo, as well as cause carbamylation of proteins, peptides, and amino acids. Other uremic toxins and their participation in the damage caused by oxidative stress to biological material are also presented. Macromolecules and molecules modified as a result of carbamylation, oxidative stress, and their adducts with uremic toxins, may lead to cardiovascular diseases, and increased risk of mortality in patients with CKD.

## 1. Introduction

Chronic kidney disease leads to uremia associated with the accumulation of uremic toxins in the body. That causes unfavorable changes in the cardiovascular system, some of them lead to chronic inflammation, endothelial dysfunction, damage to the mitochondria and, consequently, oxidative stress. In 2003, the European Uremic Toxin Work Group (EUTox) classified 90 substances as uremic toxins [1]. In 2007, the list of uremic toxins was expanded to include 14 more compounds [2]. Among the toxins, about 25 compounds show the ability of proteins binding. These compounds have been divided into several groups. Among them, there are advanced glycation end products (AGE), phenolic derivatives (including p-cresol), indole derivatives, hippurates, polyamines, peptides, homocysteine, 3-carboxy-4-methyl-5-propyl-2-furanopropionic acid (CMPF), and trimethylamine-N-oxide (TMAO). Uremic toxins can be classified according to their molecular weights and their ability to bind to proteins [3]. The most common classification divides uremic toxins into (1) low molecular weight water-soluble compounds; (2) high molecular weight compounds; and (3) protein binding compounds [1].

The toxins dissolved in uremia were classified based on their size and binding properties. Free, water-soluble compounds of low molecular weight (500 g/mol) accounted for 46% of the 88 solutes; 28% of the solutes were particles with a mass of 500–60,000 g/mol, and 24% of the solutes were associated with proteins solutes [4].

Most of them are peptides that are difficult to remove during hemodialysis unless the pore size of the dialyzer is large enough. In turn, removal of toxins associated with proteins from the body of a patient with CKD is almost impossible using dialysis [5].

Some uremic toxins such as indol acetic acid, indoxyl sulfate, p-cresyl sulfate hippuric acid, phenyl sulfate, trimethylamine N-oxide, and hydrogen sulfide in the blood of CKD patients come from the metabolism of bacteria in the gut [6,7]. As a result of the conducted research, the list of potential uremic toxins is constantly being modified. For example, it is suggested that the current list of cytokines that are considered potential uremic toxins is incomplete and excessive [8].

Many toxins presented in this review can release ROS enhancing oxidative stress in organism of CKD patients. Uremic toxins and oxidative stress lead to modifications of proteins, lipids, and DNA. Some of these modifications are protein carbonyl compounds as products of protein oxidation, lipids oxidation (thiobarbituric acid reactive substances (TBARS), 4-hydroxynonenal (4-HNE), acrolein), carbamylation, and glycation. In addition, hemodialysis worsens these processes, especially those related to oxidative stress [9].

In this review, we summarize recent research showing that uremic toxins have a biological effect on the cells and tissues of CKD patients as well as on the development of the disease. Modified biomolecules as a result of carbamylation, oxidative stress, and covalent adducts formation of uremic toxins with biopolymers can lead to cardiovascular diseases, increased risk, and mortality in patients with CKD [10,11,12].

## 2. Urea Toxicity

It is known that elevated urea levels can lead to molecular changes related to insulin resistance, generation of reactive oxygen species, apoptosis, and disruption of the intestinal protective barrier [13]. Urea is one of the earliest known uremic toxins in chronic kidney disease (CKD); its concentration in patients is about 5–7 times higher than in healthy individuals. Urea is toxic to the body at these higher concentrations because it is in equilibrium with the cyanate and 1% of the cyanate changes to the more reactive form, the isocyanate/isocyanic acid [14,15]. Isocyanic acid is also produced in the body as a result of the metabolism of thiocyanate with the participation of myeloperoxidase [16]. Isocyanic acid reacts with the thiol and amino groups of proteins, amino acids, sugars, etc. [17]. Modifications also apply to amino acids such as cysteine, histidine, arginine, lysine, valine, tryptophan, and threonine, as well as proteins containing these amino acids [18,19]. This process has been called carbamylation or carbamoylation.

Carbamylation is a post-translational modification of proteins resulting from the non-enzymatic binding of isocyanic acid to the mentioned free functional groups. This reaction has a substantial impact on the structural and functional properties of proteins and leads to an accelerated process of their aging. Carbamylation also affects such macromolecules as hemoglobin, plasma lipoproteins, albumin, membrane proteins, and erythropoietin in patients with chronic renal failure [17]. For example, carbamylated hemoglobin concentration was about three times higher in people with chronic renal failure undergoing both hemodialysis or peritoneal dialysis patients [10]. In addition, changes in the structure of hemoglobin and non-hem proteins of erythrocyte were observed in patients with CKD [11]. In turn, carbamylated low density lipoproteins (LDL) affect atherosclerotic through the participation in the formation of foam cells, induction of apoptosis of endothelial cells, and proliferation of smooth muscle cells; myeloperoxidase is involved in this process [14,20,21]. Additionally, carbamylation and oxidation in vitro led to apoptosis in lymphocytes [22]. Carbamylation causes changes in the secondary and tertiary structure of proteins, affecting the availability of the protein’s active center for enzymes. This process also leads to changes in protein–protein and/or protein–lipid interactions. For example, carbamylation of type I collagen led to disturbances in the structure of the triple helix, which reduced the polymerization capacity of normal fibers [23].

The carbamylation process is thought to be responsible for the post-translational modifications of proteins that are related to atherogenesis and other functional changes. In clinical trials, carbamylated proteins, amino acids, and other compounds have been associated with cardiovascular disease and overall morbidity and mortality [13]. It was shown that treatment of 3T3-L1 adipocytes with urea at concentrations similar to those in CKD patients led to the production of ROS, caused insulin resistance, increased the expression of adipokines, retinol-binding protein 4 (RBP4), and resistin, and increased the level of O-linked N-acetylglucosamine (O-GlcNAc) modified insulin signal molecules. In-vivo studies using uremic mice, a mouse model of surgically induced renal failure, showed ROS production, modification of insulin signaling molecules by O-GlcNAc, and an increased expression of RBP4 and resistin in visceral adipose tissue. Insulin resistance and glucose intolerance were also found in mice with uremia [24].

In vitro, after treatment of red blood cells (RBC) with urea, there was an increase in the fluidity of the lipid membrane in the polar and hydrophobic regions of the cores. The changes in the fluidity of membranes were accompanied by changes in the RBC membrane cytoskeleton. The addition of hydrogen peroxide led to the deepening of the observed changes. It seems that carbamylated proteins are more sensitive to oxidation than native proteins [25]. Carbamylated proteins may activate mesangial cells to a profibrogenic form, which may contribute to the development of renal failure [26].

One of the products of protein carbamylation is homocitrulline, which is formed by binding isocyanic acid to the ε-NH_2_ group of the side chain of lysine residues [12]. Nowadays, homocitrulline is an important biomarker in patients with uremia and its increase in concentration allows distinguishing acute from chronic renal failure and is a risk factor of mortality [27]. The observations presented here show that urea concentration in CKD patients, long considered insignificant, may lead to increased ROS production, cause insulin resistance in vitro and in vivo, and lead to cardiovascular disease and mortality in CKD patients.

In chronic kidney disease, there is a frequent disturbance of glucose homeostasis, which leads to increased mortality of patients. This likely has to do with defective insulin secretion, which may be the result of a direct action of urea on pancreatic β cells. Studies in CKD mice after nephrectomy showed abnormalities in the secretion of glucose-stimulated insulin in vivo. Similar results were observed in isolated islets. The impaired insulin secretion has also been observed in human and murine islets following administration of pathological urea concentrations found in CKD patients. In normal and urea-treated CKD mouse islets, an increase in oxidative stress and protein O-GlcNAcylation was found. These results are indicative of an impaired insulin secretion in CKD caused by elevated blood urea levels that lead to an increase in O-GlcNCylation of islet protein and impair glycolysis [28]. A good way to reduce urea levels in CKD patients is nutritional therapy with low protein content. It has been demonstrated that low protein diet may decrease urea levels and reduce protein carbamylation as well as cyanate production [18].

## 3. Phenol Derivatives

**Figure 1:**
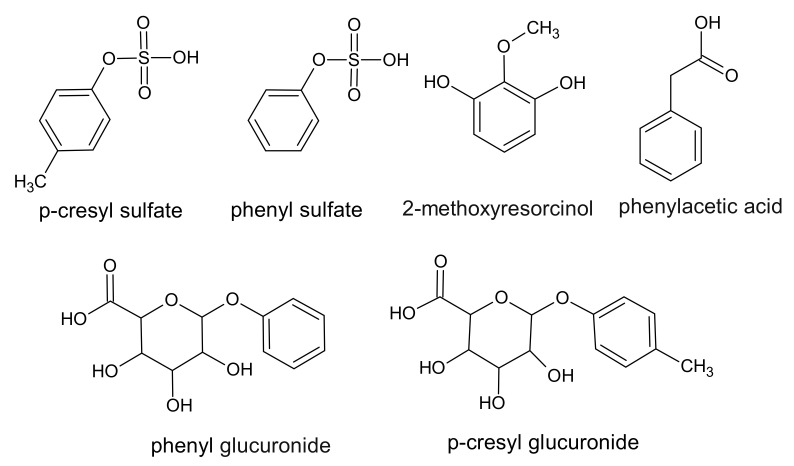
Chemical structure of phenol derivatives, classified as protein-binding uremic toxins.

Phenol derivatives are a large group of uremic toxins that bind to proteins. All phenols in humans are a product of the metabolism of phenylalanine and tyrosine by intestinal anaerobic bacteria. Chemically, phenols can be characterized as a cyclic six membered aromatic ring containing a hydroxyl group [29] (Figure 1).

The best known phenolic uremic toxin is p-cresol. However, it has been shown that after absorption in the gut, it is converted to the p-cresyl sulfate (PCS) or p-cresyl glucuronide [29,30,31]. The PCS concentration in healthy subjects is about 2.2 mg/L and in CKD patients 37.1 mg/L. During hemodialysis, for CKD patients this compound is reduced by approximately 30% compared to the baseline value [32]. Much lower concentrations are observed with p-cresyl glucuronide. In patients with CKD, they are about 5.8 mg/L, and in healthy people, they are even undetectable. Presumably, this compound during hemodialysis is reduced by 81% [32]. On the other hand, the concentration range of PCS in hemodialysis patients described by Vanholder et al. was significantly higher than that determined by Itoh et al., and it was in the range of 30–45 mg/L up to a maximum of 80–105 mg/L [31]. One of the toxic activities of PCS is the induction of oxidative stress in cells [33]. PCS, was known to be associated with the mortality rate in patients with chronic kidney disease, but the mechanism of its action was unknown. In a study by Schepers et al., a pro-inflammatory effect of PCS, as evaluated by the increased oxidative burst activity of leukocytes, was demonstrated [30]. PCS has also been shown to have a cytotoxic effect by producing ROS in renal tubular cells and initiating an increase in NADPH oxidase activity in renal tubular epithelial cells. The action of PCS led to an increase in mRNA levels, inflammatory cytokines, and the secretion of the transforming growth factor-β1 (TGF-β1) protein causing renal fibrosis [34]. Furthermore, Edamatsu et al. have observed a decrease of the total glutathione level in porcine renal tubular epithelial cell line under the influence of PCS, and it suggests that this makes cells more vulnerable to oxidative stress [35].

It was postulated that the concentration of phenol compounds in the serum of patients with CKD may increase about 4–5 times compared to healthy people; however, these assumptions are difficult to confirm [1]. Schepers et al. suggested that phenol, like p-cresol may also be in a conjugated form [29]. Itoh et al. showed that the concentration of phenyl sulfate increases approximately 18 times in people with CKD compared to healthy people and that over 90% of this compound is bound to proteins [32].

CKD is often associated with insulin resistance, but the mechanisms related to insulin resistance are poorly understood. PCS, a protein-bound uremic toxin, has been shown to be one of the causes of this phenomenon. When PCS was administered to mice with normal renal function, insulin resistance was found. Mice dosed with PCS showed altered insulin signaling in skeletal muscle through the activation of extracellular signal-regulated kinases (ERK 1/2). In addition, it was shown that treatment of C2C12 myotubes with PCS concentrations observed in CKD caused insulin resistance by direct activation of ERK 1/2. The reduction of intestinal p-cresol production by prebiotic arabinoxylooligosaccharides prevented these metabolic disorders [28].

In plasma of ESRD patients, the concentration of phenylacetic acid (PAA) is about 140 ± 45 mg/L and in the plasma of healthy subjects, the concentration of this compound was not detectable [36]. In turn, previous studies have shown that the concentration of PAA in healthy people is about 0.1 mg/L, and in patients with CKD, it is about 0.5 mg/L [32]. The decrease of this compound in serum during hemodialysis was approximately 35% [32]. Furthermore, Bohringer et al. reported the protein-bound fraction of PAA to be 59% [37]. Hence, it has been suggested that PAA can bind to various proteins in the serum, not only to albumin [38]. The role of PAA is not clear [39]. In murine macrophage cell line, it has inhibited inducible nitric oxide synthase (iNOS) mRNA and protein expression, and in mononuclear blood cells and vascular smooth muscle cells, it has induced iNOS expression [40,41]. It has also been observed that induction of iNOS expression in vascular smooth muscle cells by PAA leads to an increase in reactive oxygen species production [41].

Similarly, in human aortic endothelial cells, the ROS generation was observed in the presence of PAA. Incubation of the aortic endothelial cells with PAA has been shown to lead to an increase in 8-hydroxydeoxyguanosine (8-OHdG), which is an indicator of DNA oxidation. In addition, tumor necrosis factor-α (TNF-α) expression was observed. Administration of Tempol as a free radicals scavenger led to significant inhibition of TNF-α secretion, indicating the participation of ROS in the production of this cytokine [42]. TNF-α is a pro-inflammatory cytokine that, together with oxidative stress, performs a key role in the pathogenesis of atherosclerosis [43].

There is not much information in the literature about the toxicity of 2-methoxy resorcinol in CKD; however, various resorcinol conjugates have been reported to induce apoptosis in human pancreatic and human prostate cell lines [44].

## 4. Indole Derivatives

**Figure 2:**
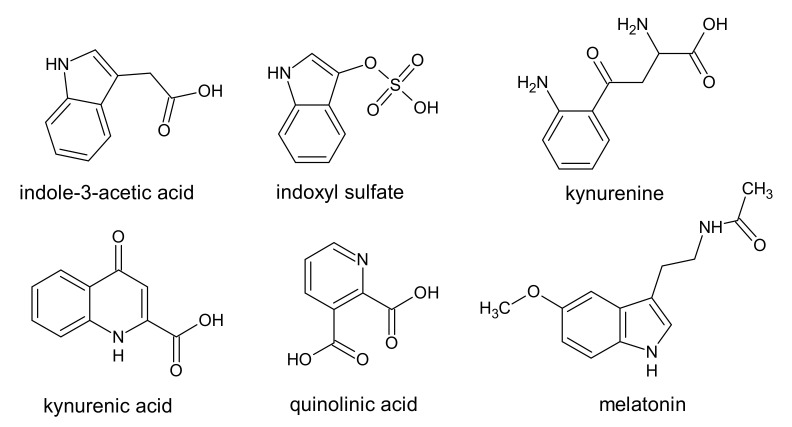
Chemical structure of indole, pyridine, and chinoline derivatives, classified as protein-binding uremic toxins.

Indoles belong to the uremic toxins with an aromatic heterocyclic structure. In the body, tryptophan can be metabolized through the indolic and the kynurenine pathways [45]. The metabolism of tryptophan in the kynurenine pathway produced intermediate metabolites such as kynurenine [39,46]. Further metabolic transformations of this compound may lead to, among others, the formation of quinolinic acid and kynurenic acid (Figure 2) [46,47]. In the gut, indole may be produced by bacteria as a product of the tryptophan degradation and then oxidized and sulfated in the liver [45]. The indolic pathway of tryptophan metabolism leads to the formation of uremic toxins: indoxyl sulfate (IS) and indole-3-acetic acid (IAA) (Figure 2) [39].

Melatonin is produced from tryptophan by the production of serotonin in the pineal gland. Although it is listed as a uremic toxin, its role in the progression of CKD is unclear [1,48]. In contrast, melatonin is supplied as an endogenous antioxidant which exhibits several unique characteristics that differentiate it from classic antioxidants. Melatonin has the ability to inhibit the cascade reaction with ROS but also the ability to induce in vivo of moderate oxidative stress. These features make melatonin a powerful antioxidant that protects organisms from increased oxidative stress [49].

Although significantly elevated levels of kynurenine, quinolinic acid, and kynurenic acid were observed in CKD patients, they do not generate much interest among researchers of uremic toxins [1]. A study of the levels of kynurenine, kynurenic acid, and quinolinic acid has shown, respectively, 2-, 11-, and 27-times higher levels of these compounds in patients with chronic kidney disease [50]. Moreover, the same studies showed a strong association of increased levels of these uremic toxins with the level of antioxidant enzymes in CKD patients. The authors suggest that the products of tryptophan metabolism in the kynurenine pathway may perform an important role in the generation of the oxidative stress, inflammation and the prevalence of cardiovascular disease in patients with end-stage renal disease in patients with CKD [51].

Indole uremic toxins such as IS, IAA, and kynurenine (a product of indole ring-opening), by activating the aryl hydrocarbon receptor (AHR), have a pro-thrombotic effect on the endothelium, especially via tissue factor induction. In addition, IS significantly increased the expression of AHR target genes PTGS2 (encoding COX2), AHRR, CYP1A1, and CYP1B1, as well as F3 (encoding TF). Loss of endothelial anticoagulant properties by toxic AHR agonists may promote a cardiovascular disease in CKD including thrombosis [52].

The most known derivative of tryptophan with toxic properties is IS. The level of this toxin in CKD patients increases about 60–80 times compared to healthy people and amounts to 30–53 mg/L in CKD and 0.5–0.6 mg/L in healthy people, respectively [1,32]. The reduction level of this compound during hemodialysis is only 30–32% and is one of the lowest similar to PCS [32]. In addition, it has been shown that about 97% of IS in the body is bound to proteins [32,33]. It has been demonstrated that the nephrotoxicity of this compound is induced by impairment of cell antioxidant systems, and by pro-inflammatory mechanisms [39] and is negatively correlated with cardiac/renal fibrosis [53,54]. Another aspect of IS toxicity is its effect on the induction of ROS production. This mechanism of IS toxicity has been well documented both in in vivo and in vitro studies [55]. Some in vitro studies have demonstrated that IS causes an increase in the production of ROS species in lymphocytes [56], erythrocytes [57], intestinal epithelial cells (IEC-6) [58], HUVEC cells [32], and human renal tubular epithelial cells (HK-2) [59]. It has been demonstrated that all the toxins listed above may have neuroactive properties [45]. It has been shown that high IS concentration led to oxidative stress and fibrosis of the heart and kidneys [53]. On the other hand, several oxidative modifications of proteins and lipids due to the presence of IS have been observed in RBC [60]. An indirect mechanism of the toxic activity of IS may be the induction of apoptosis in cells [61]. The studies discovered a novel effect of IS, i.e., the triggering of RBC shrinkage and RBC cell membrane scrambling, that may result from the induction of RBC death or eryptosis [57,62].

IS has been shown to increase the dysfunction of the vascular endothelium associated with the production of ROS due to the expression of NADPH oxidase (mainly p22phox and p47phox), mitochondrial and intracellular. Moreover, the production of ROS leads to the activation of the RhoA/ROCK pathway, which promotes the production of oxidants [63]. ROS are the main cause of endothelial dysfunction leading to vascular damage in both metabolic and atherosclerotic diseases. The first step is the activation of the endothelium, which has an abnormal pro-inflammatory and pro-thrombotic phenotype of endothelial cells. As a consequence, this leads to reduced bioavailability of nitric oxide (NO) and impaired vascular tone, as well as other phenotypic changes in the endothelium, which is collectively referred to as endothelial dysfunction [64].

Chronic kidney disease in patients is commonly associated with cardiovascular disease (CVD). Studies in rats showed that IS administration led to oxidative stress in the heart muscle, which resulted in increased levels of markers such as malondialdehyde (MDA and 8-OHdG, decreased levels of the transcription factor (Nrf2) and heme oxygenase-1 (HO-1), and decreased levels of antioxidant defense. Increased levels of TGF-β1 and type 1 collagen were also observed. Moreover, IS intensified myocardial fibrosis and cardiomyocyte hypertrophy [65].

Chronic kidney disease is accompanied by permanent inflammation and oxidative stress. White adipose tissue has been shown to be an important source of inflammation and oxidative stress. In adipocytes, the 3T3-L1 fat cells were found that IS led to the production of ROS mainly generated by the activation of NADPH oxidase. Moreover, IS increased the secretion of TNF-α and interleukin-6 by fat cells. It is likely that IS is an important mediator of adipocyte dysfunction in these patients [66].

Abnormalities in iron metabolism are observed in CKD. A key regulator of iron metabolism throughout the body is hepcidin, produced by hepatocytes. Hepcidin levels have been shown to be elevated in chronic kidney disease. Increased hepcidin levels were observed in IS-treated HepG2 cells and in an in-vivo mouse model. Adenine-induced CKD mice showed an increase in the hepcidin expression. Renal anemia, decreased plasma iron, increased serum ferritin, and increased spleen iron were observed in CKD mice. Administering IS to mice led to an increased expression of hepcidin in the liver [67].

Using the SPECT/CT imaging method with 99mTc-DTPA, an imaging marker for blood-brain barrier permeability, IS was shown to lead to cognitive impairment in the object recognition test, object localization task, and in vivo social memory tests in rats. These results indicate the activation of AHR by IS, leading to the disruption of the blood-brain barrier [68].

Another uremic toxin formed as a result of tryptophan metabolism is IAA. Indole-3-acetic acid is the most common plant hormone belonging to the auxin group. The level of this compound in people with CKD increases by about 4–4.5 times at the ratio to the level in healthy people, and during hemodialysis it is reduced by approx. 44% [32,69]. The study of Dou el al. demonstrates in vitro that IAA induces endothelial oxidative stress and demonstrates inflammatory activity [69]. Moreover, this compound is also mentioned as a potential inducer of the eryptosis process [61].

## 5. Advanced Glycation End Products (AGE)

**Figure 3:**
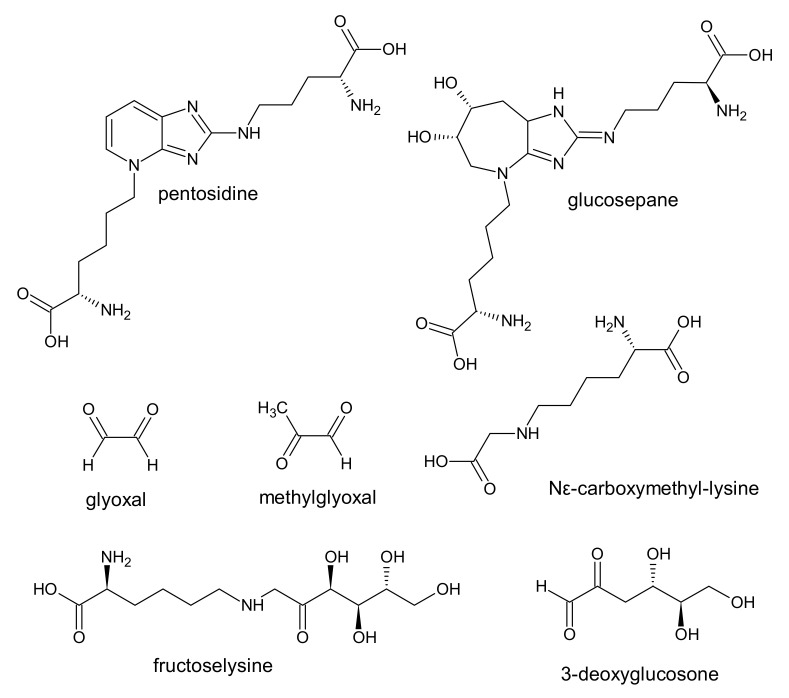
Chemical structure of some AGE, classified as protein-binding uremic toxins.

AGE result from irreversible modifications of proteins or amino acids by carbohydrates and other metabolites. The process of AGE formation is called glycation or the Maillard reaction [70]. The events responsible for their generation in uremia are oxidative and carbonyl stress. At least 20 different types of AGE have been described in the literature. Some of them, with high biological activity, are included in the list of uremic toxins (Figure 3) [1].

AGE precursors include highly reactive dicarbonyl compounds, such as glyoxal and methylglyoxal (MG). It has been shown that the levels of these compounds in CKD patients may be approximately 3.3 and 2.3 times higher, respectively, in comparison to healthy people [1]. Due to the high reactivity of carbonyl groups of glyoxal and MG and the ability to interact with diverse proteins, they were included in the group of protein-bound uremic toxins. The nucleophilic residues of proteins, such as lysine, cysteine, arginine, and histidine, are prone to react with glyoxal and MG. As a result of these reactions, among others, carboxymethyl- and carboxyethyl-lysines may occur on residues of proteins [71,72].

Important compounds contributing to the development of CKD include hydroimidazolone (MG-H1) derived from MG, Nε-carboxymethyl-lysine (CML), and glucosepane [70]. A significant precursor to AGE is the metabolite MG, which is metabolized by glyoxalase 1 (Glo1) in presence of glutathione in the cytoplasmic glyoxalase system. A hallmark of CKD is the increase in MG dicarbonyl stress, driven by decreased renal Glo1 regulation that leads to increased MG-H1 formation [70]. It was shown that MG-H1 is a dominant AGE in renal failure [73]. It is known that pro-inflammatory and pro-oxidative properties of advanced glycation end products, that accumulate in patients with CKD, may perform a major role in a high prevalence of endothelial dysfunction and subsequent cardiovascular disease [74]. Since CKD is accompanied by oxidative stress, glycoxidation adducts are more common as a result of a combination of glycation and oxidation. These compounds show higher chemical reactivity and more often cause irreversible cross-linking of proteins. Higher levels of AGE and glycoxidation products are present in people with diabetes compared to healthy people and are believed to increase oxidative stress by interacting with their receptor (RAGE), contributing to the development of vascular complications [75]. Dicarbonyls, MG, and 3-deoxyglucosone, which are highly reactive, are associated with a faster progression of CKD [76]. In turn, blood AGE levels derived from MG are associated with the histological progression of CKD, which has been documented by biopsy in type 1 diabetes. Measurement of AGE can predict loss of kidney function in diabetic kidney disease in patients with type 2 diabetes [77]. Similar conclusions, suggesting that the renal function deterioration in CKD was associated with increased AGE levels, were made by Tezuka et al. [78].

## 6. Hippurates

**Figure 4:**
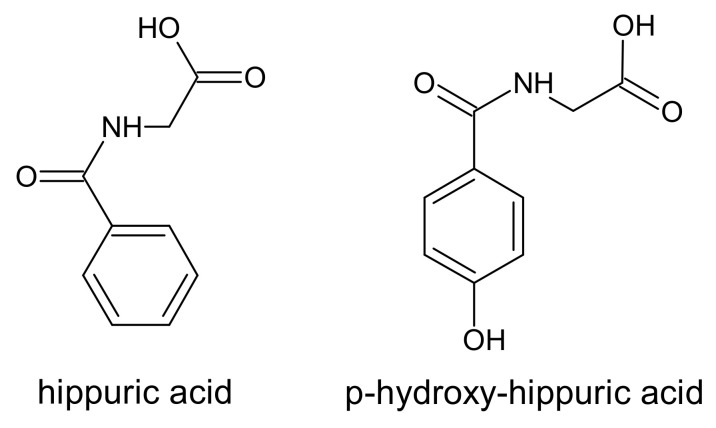
Chemical structure of hippurate derivatives, classified as protein-binding uremic toxins.

Two N-benzoylglycine derivative compounds, called hippurates, belong to the group of protein-binding uremic toxins. The name hippurates comes from the Greek word for horse because it was in the urine of these animals that hippuric acid was originally identified [79]. Both hippuric acid and p-hydroxy-hippuric acid (Figure 4) are normal components of urine with a strong association with diet and the intestinal microbiota. In the gut, phenylalanine, quinic acid, and various phenolic compounds are metabolized by microbiota to phenylpropionic acids. In the liver, by the β-oxidation, phenylpropionic acid is metabolized to benzoic acid, and then the latter is conjugated with glycine to hippuric acid [80]. p-hydroxy-hippuric acid is a derivative of hippuric acid with an attached hydroxy group in a para position of the benzene.

In patients with ESRD, the concentration of p-hydroxy-hippuric acid increased on average to the level of 18.3 ± 2.3 mg/L [81]. However, the authors of the study were not able to accurately determine the level of p-hydroxy-hippuric acid in healthy people due to the low sensitivity of the used proton nuclear magnetic resonance spectroscopy (H-NMR) method [81]. Therefore, the concentration of p-hydroxy-hippuric acid was apparently lower than 1.17 mg/L, the limit of detection of 500 MHz H-NMR spectrometer. This suggests that the level of p-hydroxy-hippuric acid may be several times higher in CKD patients in comparison to healthy people. Moreover, Jankowski et al. show that during blood dialysis in ESRD patients, the level of this toxin is reduced by only 53% [81].

In in vitro experiments exploring the effect of p-hydroxy-hippuric acid, it demonstrated its inhibition action of Ca^2+^-ATPase in the plasma membrane of erythrocytes from healthy volunteers [81]. The authors suggest that only a part of this compound may penetrate the plasma membrane and intracellular space to inhibit Ca^2+^-ATPase, because of its protein binding properties. The disturbances in intracellular calcium levels can result in the induction of apoptosis. Unexpectedly, p-hydroxy-hippuric acid inhibited apoptosis of polymorphonuclear leukocytes and diminished the activities of caspases 3, 8, and 9 [82]. Moreover, it has been demonstrated that the presence of hippuric acid and p-hydroxy-hippuric acid correlated negatively with motor nerve conduction velocities [83].

Accumulation of hippuric acid has been shown to be associated with disease progression in patients with CKD. The average total concentration of this acid in patients with CKD is approx. 71.3 mg/L, and in healthy subjects approx. 3 mg/L. This means that the level of hippuric acid in CKD patients can be almost 24 times higher than in healthy people [4]. Itoh et al. suggest that its reduction rate by HD is more than 68% [32]. The hippuric acid can lead to kidney fibrosis. One of the most important factors influencing nephrotoxicity is oxidative stress. The hippuric acid promoted renal fibrosis by interfering with redox homeostasis in HK-2 (human renal tubular epithelial cells) cells, increased gene expression associated with fibrosis, extracellular matrix imbalance and oxidative stress. Moreover, hippuric acid disrupted antioxidant systems by lowering the level of factor 2 associated with erythroid factor 2 (NRF2), leading to ROS accumulation [84].

It has been demonstrated that p-hydroxy-hippuric acid may induce free radical production in the renal proximal tubular cell line, opossum kidney (OK) cells, transformed with hOAT1. The hippuric acid did not effect on the production of free radicals in cells [85]. On the other hand, in HUVEC cell line, in the presence of hippuric acid (144.8 mg/L), ROS production significantly increased [32].

## 7. Polyamines

**Figure 5:**
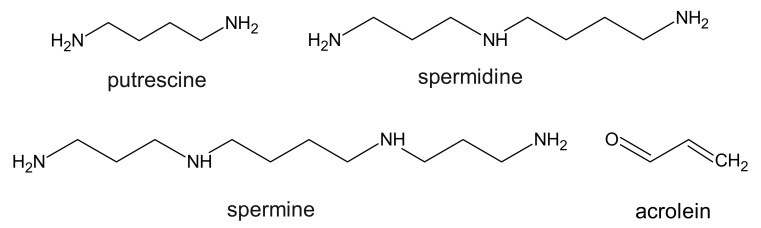
Chemical structure of polyamines and their derivative, classified as protein-binding uremic toxins.

The research carried out in 1983 showed that in patients with CKD, the level of polyamines (putrescine, spermine, and spermidine) (Figure 5) increases significantly in comparison to healthy volunteers [86].

Vanholder et al. have classified polyamines among the group of protein-binding uremic toxins [1]. However, it is suggested that those tests may have been biased due to a faulty measurement method [87]. The problem concerns elevated levels of spermidine in patients with CKD and their toxic effects on cells. Indeed, significantly higher levels of putrescine in the plasma of CKD patients were shown, but the levels of spermine and spermidine were lower than in healthy subjects. Putrescine showed an antioxidant effect in the cells by alleviating oxidative stress induced by antibiotics or H_2_O_2_. In contrast, putrescine induced the apoptotic process associated with the release of cytochrome c from the mitochondria and activation of caspase cascades, with the consequent cleavage of caspase-2, polyA-ribose polymerase (PARP), and proteolytic cleavage of translation initiation factor 4G (eIF4G) [88]. However, in patients with CKD, the polyamine oxidase activity was over 2 times higher than in normal people [87,89]. It has been suggested that in serum, spermine and spermidine are metabolized by amine oxidase to aminoaldehyde or aminodialdehyde with the evolution of H_2_O_2_ and NH_3_. Then, as a result of the transformation of amino aldehydes, acrolein is formed (Figure 5) [87,89]. The level of acrolein is quite varied and amounts to approx. 17–39 µg/L in healthy people and 50–84 µg/L in patients with CKD [89,90,91], which means that in CKD patients, the level of free acrolein was 3–5-fold higher when compared to control subjects. Moreover, it has been shown that the average level of this compound is just 32% values lower in post dialysis samples [90]. The authors also suggest that oxidative stress in patients with CKD may contribute to an increase in acrolein production during hemodialysis, and thus reduce the kinetics of its removal [90]. On the other hand, a recent report demonstrated that in non-hemodialyzed patients, the levels of acrolein and protein-conjugated acrolein were even higher than in hemodialyzed patients [92]. Moreover, the increase in protein-conjugated acrolein was strongly correlated with the increase in creatinine level in serum. Furthermore, it has been reported that acrolein and H_2_O_2_ produced from spermine are involved in cell damage and that acrolein may also cause lipid peroxidation of erythrocytes [93]. Some research shows that polyamines concentration (putrescine, spermine, and spermidine) negatively correlated with erythropoiesis in CKD patients [94]. This means that polyamines may be strongly associated with anemia in patients with CKD. The conducted research shows that the toxicity of acrolein to cells was 10-fold greater than that of H_2_O_2_ [95]. Considering the fact that polyamines are necessary for cellular growth, some research suggests that it is in fact acrolein and not polyamines, that functions as a uremic toxin in patients with CKD. Acrolein, produced mainly from spermine, is a powerful poison. Many diseases such as ischemic stroke, dementia, Sjögren’s syndrome, and kidney failure are related to acrolein. A protein-conjugated acrolein (PC-Acro) marker with high sensitivity and specificity along with IL-6 and C-reactive proteins has been found in plasma ischemic stroke and dementia. The level of PC-Acro in plasma and saliva was positively correlated with the severity of the renal failure and Sjgren’s syndrome, respectively [92].

The glutathione (GSH) is the first line of defense against acrolein-induced toxicity. In high doses of acrolein, the concentration of GSH is low to provide cellular protection against this toxin [96]. Its reaction with the cysteine thiol group was about 110–150 times faster than HNE, a famous product of lipid peroxidation. Given its high affinity for thiols, it could be possible to lower its concentration by administering N-acetlcysteine or other thiols used in the treatment of heavy metal poisoning. Acrolein toxicity is related to the formation of covalent adducts of DNA and proteins via cysteinyl, histidyl, and lysyl residues as well as free N-terminal amino groups [97,98].

## 8. Other Compounds

**Figure 6:**
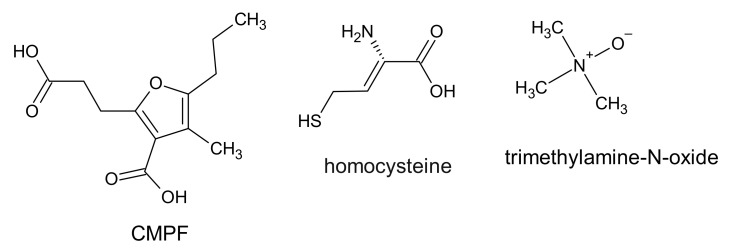
Chemical structure of CMPF, homocysteine, and trimethylamine-N-oxide, classified as protein-binding uremic toxins.

To the protein-bound uremic toxins belongs the 3-carboxy-4-methyl-5-propyl-2-furan propionic acid (CMPF), which is a metabolite of furan fatty acid and a marker of fish oil intake. The CMPF chemical structure is based on a furan dicarboxylic acid derivative (Figure 6).

The origin of CMPF is not clear; however, it has been suggested that some furanoid acids can be precursors of CMPF. Those acids are found in food such as fish, vegetables, and fruit. It cannot be excluded that CMPF is also produced de novo in the human body [99]. CMPF is a metabolite which is present in plasma at high concentrations in patients with type 2 diabetes and CKD patients. CMPF inhibited insulin secretion in mouse and human islets in vitro and in vivo in rodents [100]. The serum concentration of CMPF is about 4 mg/L in healthy people and 21 ±1.3 mg/L in patients with CKD before hemodialysis. The level of CMPF in serum was significantly higher after hemodialysis (27.3 ±1.8 mg/L) compared to pre-hemodialysis [32]. In contrast, it is known that the concentration of CMPF in plasma does not change before and after hemodialysis [101,102]. The ratio of CMPF binding to proteins is nearly 100% and this bond is stronger than for IS or PCS [99]. For this reason, CMPF cannot be removed by conventional hemodialysis. CMPF has been involved in the inhibition of drug binding to serum albumin and mitochondrial respiration in cells [32,99]. Moreover, CPMF, in the presence of albumin, was involved in the production of ROS in HUVEC cell lines [32]. However, the role of CMPF as a uremic toxin remains controversial. Recently, Luce and colleagues showed that the accumulation of CMPF was not associated with metabolic disturbances and increased mortality in patients with CKD. The authors, therefore, suggest that CMPF should not be considered as a uremic toxin. Indeed, they believe that it is an indicator associated with better nutritional status and may be a marker of a healthy diet and omega 3 consumption. Its elevated serum levels in CKD patients were not associated with mortality and cardiovascular disease [102].

Homocysteine is a thiol-containing amino acid created as a result of methionine metabolism. The serum concentration of homocysteine in CKD patients is about 3–4 times higher in comparison to healthy people. This amino acid contains a highly reactive thiol group and can undergo oxidation in the presence of oxygen and metal ions generating oxidative stress in cells. It has been demonstrated that homocysteine has a causal role in the development of CKD. The mechanisms of its action in the cells include, among others, oxidative stress, inflammation, and DNA hypomethylation [103]. Additionally, elevated levels of homocysteine (hyperhomocysteinemia) are linked to various diseases such as homocystinuria, neurodegenerative diseases, and liver disease. In experiments carried out on rats, it was shown that hyperhomocysteinemia decreased the antioxidant defense and led to a decrease in thiols, and increased lipid peroxidation in the rat liver, which is indicative of oxidative stress. Liver damage caused by homocysteine may explain, in part, the mechanisms of liver disease associated with hyperhomocysteinaemia [104].

The microbes in the gut produce various substances such as trimethylamine, short chain fatty acids, and secondary bile acids, which can contribute to cardiovascular disease. Trimethylamine produced by bacteria is oxidized to trimethylamine-N-oxide (TMAO) by flavin-containing monooxygenase [6]. This enzyme is expressed in the liver and oxidizes xenobiotics containing amines or sulfides [105]. A significantly higher level of this compound was observed in the plasma of CKD patients (30.33 μmol/L) in comparison to the level in healthy subjects (2.08 μmol/L) [106]. The higher circulating TMAO blood levels in patients with ESRD and CKD compared to patients without CKD were also observed by Bain et al. [107]. Moreover, it has been suggested that a higher level of TMAO may precede overt renal failure [6].

Table 1 summarizes and specifies the percentage of protein binding and reduction during hemodialysis for each described in the text uremic toxin.

## 9. Implications of Uremic Toxins and Oxidative Stress in Cardiovascular Disease

In recent years, ROS that initiate oxidative stress have been shown to perform a key role in the development and maintenance of inflammation, contributing to the pathophysiology of many debilitating diseases such as cardiovascular diseases (CVD), diabetes, cancer or neurodegenerative diseases [109]. ROS affect all stages of the inflammatory response, releasing molecules from damaged tissues that act as endogenous signals of danger. These signals are recorded by the innate immune receptors Toll (TLR) and NOD-like (NLR) and activate signaling pathways that initiate an adaptive cellular response to such signals [110].

**Figure 7:**
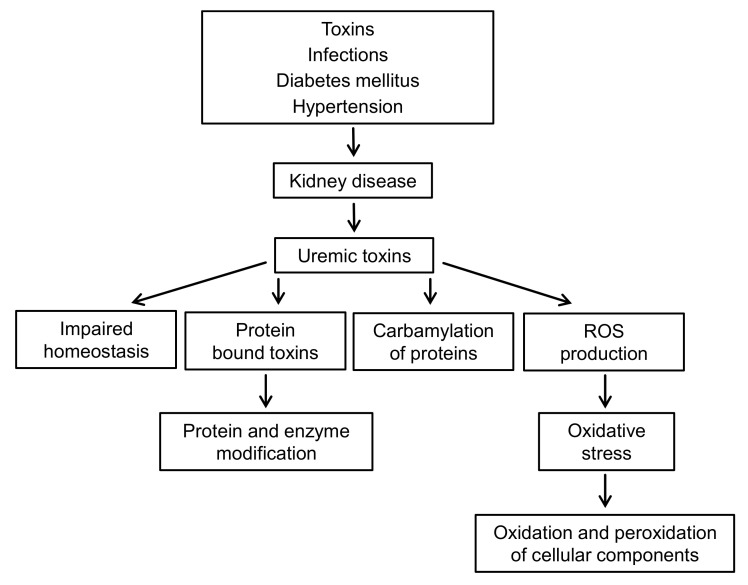
Cells and tissue damage in chronic kidney disease.

CKD is accompanied by oxidative stress and chronic inflammation [111]. Inflammation is caused by a variety of factors, such as increased production and decreased clearance of inflammatory cytokines, oxidative stress, acidosis, chronic and recurrent infections, also associated with dialysis. Figure 7 presents the complexity of the interactions between uremic toxins and oxidative stress in patients with CKD.

Inflammation is directly related to the glomerular filtration rate (GFR) in dialysis patients, where it eliminates extracorporeal factors such as contamination in dialysis water, microbiological quality of dialysate, and biocompatibility of membranes in dialyzers [112]. Uremic toxins and ROS promote inflammation and oxidative stress, among others by stimulating polymorphonuclear lymphocytes, leading to the release of inflammatory cytokines: interleukins (IL) -1β and IL-8, and TNF-α as well as to the stimulation of the innate immune response by CD8 + cells [113,114,115]. In turn, oxidative stress is the result of the overproduction of ROS and lowered antioxidant defense. This applies to both the enzymatic defense related to the activity of enzymes such as superoxide dismutase (SOD1), catalase (Cat), glutathione peroxidase (GPx), and low molecular weight antioxidants, e.g., glutathione, ascorbic acid, α-tocopherol, β-carotene, ubiquinol, bilirubin, and others. In CKD, an impaired antioxidant system is observed, which is associated with decreased activity of GPx and Cat in the plasma, and increased activity of GPx and SOD1 (CuZnSOD) in RBC [116,117]. In other studies, decreased glutathione peroxidase activity and decreased SOD1 activity were observed [118]. The activity of GPx is influenced by the decreased concentration of selenium in the blood and kidney disease. Another marker of antioxidant activity was paraoxonase, which is an esterase enzyme related to high density lipoproteins (HDL) and works to protect LDL and HDL from oxidation. Paraoxonase and GPx activity was decreased in patients with renal failure, while an increase in F2-isoprostanes (prostaglandin (PG) F(2)-like compounds formed via non-enzymatic peroxidation of arachidonic acid) was observed [119].

An additional problem in CKD patients is type 2 diabetes, which is a significant cause of kidney damage. Diabetic nephropathy occurs in more than 40% of people with diabetes and performs a major role in end-stage renal disease. Various factors influence the development and progression of diabetic nephropathy. Hyperglycemia leads to overproduction of ROS, which in turn causes oxidative stress, which is an important factor in the pathogenesis of diabetic nephropathy [120]. Moreover, in CKD, there is a high concentration of uric acid in the plasma (hyperuricemia). Uric acid synthesis can promote oxidative stress directly through the activity of xanthine oxidoreductase [121]. This enzyme is synthesized as xanthine dehydrogenase and is converted to xanthine oxidase [122]. In addition to the ROS sources listed, large amounts of oxidants are produced during hemodialysis of CKD patients. The low biocompatibility of dialysis membranes leads to the activation of neutrophils and a respiratory burst in which superoxide anion radical, hydrogen peroxide, hydroxyl radicals, singlet oxygen, nitric oxide, and hypochlorous acid are produced [123]. Nitric oxide is a precursor to other reactive forms, such as nitrogen dioxide and peroxynitrite, a compound with a strong oxidizing potential [124].

Impaired glomerular filtration of the kidneys leads to the retention of toxic solutes that affect all organs of the body. The consequences of kidney dysfunction are CVD and infections, which increase the morbidity and mortality of patients with CKD. Both causes are directly or indirectly related to the weakened immune defense. The roles of cells such as polymorphonuclear leukocytes, monocytes/macrophages, lymphocytes, and antigen presenting cells in maintaining an effective immune response are disrupted. As a result, their normal response may be impaired, leading to infectious diseases or CVD [125].

Uremic toxins, oxidative stress, and permanent inflammation lead to a CVD in patients with CKD [126]. Both CVD and CKD, the so-called ”cardiorenal or renocardiac syndrome”, are directly associated with increased cardiovascular morbidity and mortality [127]. About half of all deaths of CKD patients are thought to be a direct consequence of CVD. Successful kidney transplantation significantly reduces the risk of death compared to long-term dialysis treatment, but also in this group of patients, the incidence of CVD remains high and amounts to approximately 20–35% of mortality in patients with kidney transplants [128]. It is noteworthy that a particular role in reducing the progression of CKD and related CVD is assigned to the low protein and high fiber nutritional therapy. [18,126].

Uremic cardiomyopathy is associated with the pathology of the heart that is common in CKD. CKD patients have cardiac arrhythmias, especially atrial fibrillation. CKD often causes changes in the structure and/or function of the heart referred to as “uremic cardiomyopathy” (decreased left ventricular contraction/expansion function leading to heart failure) [129]. Initially, this condition is associated with left ventricular hypertrophy (LVH) and the characteristic fibrosis of the heart muscle, thickening of the wall of arterioles inside the heart muscle, and diastolic dysfunction, followed by systolic dysfunction, leading to overt heart failure at a later stage [130]. LVH is a frequently used surrogate marker for uremic cardiomyopathy to assess the possibility of cardiovascular death. Interestingly, as many as 75% of hemodialysis patients develop LVH in the early stages, leading to a high risk of cardiac death [131]. Moreover, patients with CKD have a predisposition to arrhythmias with an increased risk of sudden cardiac death [129,132]. Some uremic toxins, including IS, PCS, fibroblast growth factor-23, and asymmetric dimethylarginine, induce direct cardiotoxicity [127]. An additional risk factor for vascular diseases in CKD is hyperuricemia, which is associated with coronary artery disease, left ventricular hypertrophy, atrial fibrillation, myocardial infarction, and ischemic stroke [133,134,135].

In patients with CKD, accelerated atherosclerosis and reduced bioavailability of nitric oxide in vascular tissues are observed. LDL is rapidly oxidized and the transformation of monocytes into foam cells is enhanced. The next stage is the proliferation of vascular smooth cells and the production of inflammatory cytokines. The overproduction of ROS in CKD promotes reduced bioavailability of nitric oxide, which quickly reacts with the superoxide anion to form peroxynitrite, which in turn leads to oxidative stress and endothelial cell dysfunction, and consequently to atherosclerosis. Moreover, in CKD, the balance between pro and anti-inflammatory factors and between pro and anti-apoptotic factors is disturbed [125]. It has been shown that LDL isolated from CKD patients is more susceptible to peroxidation in vitro than LDL from healthy subjects [136]. Soluble uremic toxins such as guanidine, PCS, IS, and asymmetric dimethylarginine have been suggested to be pro-atherogenic in CKD patients [137].

It has been shown that TMAO may be an independent risk factor for cardiovascular disease as well as other disorders [138,139]. Moreover, patients with high levels of TMAO in plasma are at increased risk of myocardial infarction or stroke [140]. The pathophysiological role of TMAO may extend not only to in-creasing cardiovascular risk, but also to the progression of renal dysfunction [6]. CKD patients have significantly higher rates of the diagnosis of sepsis compared to patients without diagnosed CKD. Bacterial infections are the leading cause of hospitalization of CKD patients and are related to vascular access, which is the most common source of bacteremia in hemodialyzed patients. Sepsis is associated with an increased risk of thrombosis, myocardial infarction, congestive heart failure, stroke, and peripheral arterial disease [105,141].

Recently, it has been shown that the mortality rate of COVID-19 is higher in the group of patients with chronic kidney disease. This also applies to the acute kidney injury from COVID-19 infection [142].

## 10. Conclusions

The list of all chemical compounds classified as uremic toxins includes over 100 items, which have been divided into three groups. More than 25 of these are classified as protein-binding uremic toxins. Chronic kidney disease is characterized by the accumulation of uremic toxins due to inadequate kidney function. These accumulated compounds due to their toxicity have a negative impact on biological functions and exert pathological effects on immune systems, kidneys, blood vessels, and the heart. The conducted research provides more information on the mechanisms of their action in cells. It often turns out that the list of uremic toxins and their mechanisms of action is modified as research progresses and knowledge about them is acquired. The presented work is an attempt to review the latest literature on protein-binding uremic toxins. However, the authors are aware that the discussed topic has not been exhausted.

The goal of hemodialysis is to clear the blood of uremic toxins, unfortunately, the protein-bound toxins are not completely removed. On the other hand, this route removes many valuable compounds with low molecular weight, such as vitamins, hormones, and others. It seems that future research should be aimed at supplementing these most valuable lost compounds after each hemodialysis session, which would certainly result in the improvement of the condition of hemodialyzed patients.

## Figures and Tables

**Table 1:** The percentage of protein binding and reduction during hemodialysis for each described uremic toxin.

Uremic Toxin	~% Reduction Rate during HD	Reference	~% Protein Binding	Reference
3-deoxyglucosone	72	[73]		
acrolein	30–32	[90,93]		
AGE (total)	45–56	[108]	50	[108]
CMPF	−30 −17–(−10)	[32] [99,108]	100	[32,99]
glyoxal	45	[73]		
hippuric acid	68–70	[32,108]	33–48	[32,108]
indole-3-acetic acid	42–47	[32,69,108]	82–94	[32,69,108]
indoxyl glucuronide	80	[32]	60	[32]
indoxyl sulfate	25–33	[32,99,108]	90–98	[32,33,37,99,108]
kynurenic acid	35	[50]		
kynurenine	15	[50]		
methylglyoxal	69	[73]		
Nε-carboxymethyl-lysine	85	[73]		
p-cresyl sulfate	29	[32]	93–97	[32,33,37,99]
p-cresyl glucuronide	81	[32]		
pentosidine	69	[73]		
phenyl glucuronide	69	[32]	76	[32]
phenyl sulfate	65	[32]	91	[32]
phenylacetic acid	35	[32]	59–60	[32,37]
p-hydroxy-hippuric acid			53	[81]
quinolinic acid	61	[50]		

## Data Availability

Not applicable.

## Abbreviations

4-HNE	4-hydroxynonenal
8-OHdG	8-hydroxydeoxyguanosine
AGE	advanced glycation end products
AHR	aryl hydrocarbon receptor
Cat	catalase
CKD	chronic kidney disease
CMPF	3-carboxy-4-methyl-5-propyl-2-furanopropionic acid
ERK ½	extracellular signal-regulated kinases
ESRD	end-stage renal disease
GFR	glomerular filtration rate
GPx	glutathione peroxidase
HDL	high density lipoproteins
H-NMR	proton nuclear magnetic resonance
IL-18	Interleukin-18
iNOS	nitric oxide synthase
IS	indoxyl sulfate
LDL	low density lipoproteins
LVH	left ventricular hypertrophy
MDA	malondialdehyde
O-GlcNAc	O-linked N-acetylglucosamine
PAA	phenylacetic acid
PC-Acro	protein conjugated acrolein
PCS	p-cresyl sulfate
RBC	red blood cells
RBP4	retinol-binding protein 4
ROS	reactive oxygen species
SOD1	superoxide dismutase
TBARS	thiobarbituric acid reactive substances
TGF-β1	transforming growth factor-β1
TMAO	trimethylamine-N-oxide
TNF-α	tumor necrosis factor-α
